# Genomic Landscape Reveals Chromosomally-Mediated Antimicrobial Resistome and Virulome of a High-Risk International Clone II *Acinetobacter baumannii* AB073 from Thailand

**DOI:** 10.1155/2024/8872463

**Published:** 2024-04-30

**Authors:** Rattiya Cheewapat, Jadsadaporn Redkimned, Sirikran Lekuthai, Thawatchai Kitti, Kannipa Tasanapak, Aunchalee Thanwisai, Sutthirat Sitthisak, Thanet Sornda, Hathaichanok Impheng, Sudarat Onsurathum, Udomluk Leungtongkam, Supaporn Lamlertthon, Siriwat Kucharoenphaibul, Jintana Wongwigkarn, Pantira Singkum, Pattrarat Chanchaithong, Rapee Thummeepak

**Affiliations:** ^1^Department of Microbiology and Parasitology, Faculty of Medical Science, Naresuan University, Phitsanulok 65000, Thailand; ^2^Department of Oriental Medicine, Chiang Rai College, Chiang Rai 57000, Thailand; ^3^Department of Biochemistry, Faculty of Medical Science, Naresuan University, Phitsanulok 65000, Thailand; ^4^Department of Physiology, Faculty of Medical Science, Naresuan University, Phitsanulok 65000, Thailand; ^5^Department of Microbiology and Immunology, Faculty of Tropical Medicine, Mahidol University, Salaya 10400, Thailand; ^6^Department of Veterinary Microbiology, Faculty of Veterinary Science, Chulalongkorn University, Bangkok 10330, Thailand; ^7^Centre of Excellence in Medical Biotechnology, Faculty of Medical Science, Naresuan University, Phitsanulok 65000, Thailand

## Abstract

This study utilized integrative bioinformatics' tools together with phenotypic assays to understand the whole-genome features of a carbapenem-resistant international clone II *Acinetobacter baumannii* AB073. Overall, we found the isolate to be resistant to seven antibiotic classes, penicillins, *β*-lactam/*β*-lactamase inhibitor combinations, cephalosporins, carbapenems, aminoglycosides, fluoroquinolones, and folate pathway antagonists. These resistance phenotypes are related to various chromosomal-located antibiotic resistance determinants involved in different mechanisms such as reduced permeability, antibiotic target protection, antibiotic target alteration, antibiotic inactivation, and antibiotic efflux. IC2 *A. baumannii* AB073 could not transfer antibiotic resistance by conjugation experiments. Likewise, mobilome analysis found that AB073 did not carry genetic determinants involving horizontal gene transfer. Moreover, this isolate also carried multiple genes associated with the ability of iron uptake, biofilm formation, immune invasion, virulence regulations, and serum resistance. In addition, the genomic epidemiological study showed that AB073-like strains were successful pathogens widespread in various geographic locations and clinical sources. In conclusion, the comprehensive analysis demonstrated that AB073 contained multiple genomic determinants which were important characteristics to classify this isolate as a successful international clone II obtained from Thailand.

## 1. Introduction

One of the problematic pathogens belonging to the ESKAPE (*Enterococcus faecium*, *Staphylococcus aureus*, *Klebsiella pneumoniae*, *Acinetobacter baumannii*, *Pseudomonas aeruginosa,* and *Enterobacter* spp.) is *A. baumannii* due to its ability to “escape” killing by antibiotics and its high burden of healthcare-associated infections (HAIs) [1, 2]. According to the WHO priority pathogens list, carbapenem-resistant *A. baumannii* (CRAB) is also designated as a critical priority pathogen for encouraging the research and development of new antimicrobials [3, 4]. HAIs caused by *A. baumannii* include ventilator-associated pneumonia, bloodstream, skin, and urinary tract infections, and secondary meningitis [5].

Molecular epidemiology investigations have classified nine international clonal lineages (IC1-9) of *A. baumannii*, which represent genetically distinct populations spreading in various geographic locations [6–8]. The ICs distributed worldwide comprise three major clones (IC I, II, and III) also known as global clones (GC I, II, and III) [6, 7]. Like in European countries, IC2 was reported as a dominant clone that successfully spread in many regions of Thailand [7, 9, 10]. *A. baumannii* is an important carrier of antibiotic resistance genes located on both chromosomal or plasmid DNA, which can be exchanged by horizontal transfer or multiplied by clonal expansions [7]. The majority of virulence factors found in *A. baumannii* are capsular polysaccharides, outer membrane proteins (OMPs), protein secretion systems, lipopolysaccharides (LPS), phospholipases, proteases, and iron-acquisition systems [11].

Whole-genome sequencing (WGS) technologies can be applied to monitor public health surveillance and molecular epidemiology along with putative mechanisms involving virulence and drug resistance [12]. In this study, we aimed to use the whole-genome sequence to understand genomic-based epidemiology, and identify genes associated with antimicrobial resistance and pathogenicity of an IC2 *A. baumannii* AB073 isolated from Thailand.

## 2. Materials and Methods

### 2.1. Bacterial Strain and Species Identification

The clinical isolate of *A. baumannii* strain AB073 was obtained from the tertiary hospital in lower northern Thailand in 2013. The AB073 was isolated from a sputum sample of a 77-year-old woman patient with ventilator-associated pneumonia in the medical ward. This isolate was presumptively identified to the genus level by biochemical testing such as Gram stain, motility test, catalase test, oxidase test, and lactose-fermenting test as previously recommended [13]. We then identified two species using the VITEK 2 system (bioMérieux. Marcy l'Etoile, France), and a partial *rpoB* sequencing was conducted as previously documented [14]. The isolate was stored at −40°C in Luria–Bertani (LB) broth (HiMedia, India) containing 20% glycerol.

### 2.2. *In Vitro* Antimicrobial Susceptibility Testing

The disc diffusion by Kirby–Bauer technique [15] was used to determine the antibiotic resistance profile following the recommendations and interpretations of the Committee for Clinical and Laboratory Standards Institute (CLSI) (2020) [16] for fifteen common antibiotics. The antibiotic-containing discs (HiMedia, India) used in this study were selected from various classes of antibiotics such as ampicillin (30 *μ*g/disc), sulbactam/cefoperazone (105 *μ*g/disc), imipenem (10 *μ*g/disc), meropenem (10 *μ*g/disc), doripenem (10 *μ*g/disc), cefotaxime (30 *μ*g/disc), ceftazidime (10 *μ*g/disc), ceftriaxone (30 *μ*g/disc), cefepime (30 *μ*g/disc), amikacin (30 *μ*g/disc), gentamicin (30 *μ*g/disc), ciprofloxacin (5 *μ*g/disc), levofloxacin (5 *μ*g/disc), trimethoprim/sulfamethoxazole (25 *μ*g/disc), tetracycline (30 *μ*g/disc), and piperacillin/tazobactam (110 *μ*g/disc). The minimum inhibitory concentrations (MICs) for imipenem and tigecycline (Sigma-Aldrich Chemicals Pvt. Ltd, India) were performed by the broth dilution method. For colistin (Sigma-Aldrich Chemicals Pvt. Ltd, India), we performed both broth and agar dilution methods as previously recommended [17]. *Escherichia coli* ATCC25922 was used as a quality control strain. EUCAST breakpoints were used for the interpretation of colistin MIC results (susceptible, ≤2 *μ*g/ml; resistant, >2 *μ*g/ml). The MIC results were interpreted using the US FDA tigecycline susceptibility breakpoints for Enterobacteriaceae (susceptible: ≤2 *μ*g/ml; resistant: ≥8 *μ*g/ml). In the case of imipenem, FDA MIC breakpoints for imipenem tested against *A. baumannii* (susceptible: ≤2 *μ*g/ml; resistant: ≥8 *μ*g/ml) were applied in this study [18] Resistance phenotypes including MDR, CR, and XDR were classified according to the previously published report [19]. The conjugation assays were carried out in the LB medium using two recipient strains, NU13R and NU15R, as described previously [20].

### 2.3. Whole-Genome Sequencing and General Genome Analysis

The total genomic DNA sample of *A. baumannii* strain AB073 was extracted and purified using a Wizard® Genomic DNA Purification Kit (Promega, USA). The purified genomic DNA was quantified and qualified using an Agilent® 2100 Bioanalyzer, (Agilent Technologies, Inc., Santa Clara, CA, United States). The DNA library was generated by following the Nextera XT DNA Library Prep Kit Reference Guide prior to paired-end sequencing on a MiSeq sequencer, according to the manufacturer's instructions (Illumina). The raw reads were subjected to quality trimming, de novo assembling, and assembled-contig correcting by using bioinformatics software, Trim Galore v0.6.7 [21], Unicycler v0.4.8 [22], and Pilon v1.23 [23], respectively. To identify the relative genomes of AB073, a similar genome finder was conducted [24]. The resulting contigs were ordered in the Multi-CSAR web server [25] by using the relative genomes of AB073 as references.

### 2.4. Downstream Bioinformatics Analysis

The ordered chromosomal DNA sequence of AB073 was subjected to detections of acquired antimicrobial resistance genes using ABRicate v0.8 [26] against the ResFinder [27]. Virulence factors were identified by using the VF analyzer part of the Virulence Factors of Pathogenic Bacteria (VFDB) database using the default settings [28]. The types of bacterial surface polysaccharide locus were classified using Kaptive v2.0 [29]. MLST and core-genome MLST (cgMLST) types were identified using MLST v2.22 [30] against PubMLST Pasteur and Oxford schemes and cgMLSTFinder [31], respectively. Insertion sequences were detected using MobileElementFinder [32]. Plasmid typing was conducted as previously described [33, 34]. A comprehensive analysis was performed with rMAP [35] to study the complete antimicrobial resistome and virulome of AB073 and a selected set of *A. baumannii* obtained from Thailand.

GrapeTree v1.5.0 was used for visualizing cgMLST-based strain clusters among AB073 and 2,648 *Acinetobacter baumannii* genomes retrieved from the PubMLST database [36]. For pangenome analysis, the ordered chromosome of AB073 and its closely related chromosomes were circularized with Circulator v1.5.1. [37] using dnaA as a marker. These circularized genomes were subjected to reannotation using Prokka v1.13.3 [38]. The annotated genes among selected strains were subjected to Roary v3.12 [39] for identifying core genes. Resulted core genes were used to compute numbers of single nucleotide polymorphisms (SNPs) between AB073 and its relatives using SNP-dists v0.7.0 [40]. In silico identifications of mobilome were conducted using two available softwares, MobileElementFinder [32] and oriTfinder [41].

## 3. Results

### 3.1. Isolate Identification and Antibiotic Resistance of *A. baumannii* AB073

The clinical *A. baumannii* strain AB073 was identified as *Acinetobacter* spp. based on the biochemical characteristics (Gram-negative, nonmotile, catalase-positive, oxidase-negative, and nonlactose fermenting). Partial sequence analysis of *rpoB* revealed that AB073 *rpoB* was highly related to the *rpoB* of two *A. baumannii*-type strains, ATCC19606 (QFQ03811.1) and ATCC17978 (A1S_0287), with similarity values of 99.6 and 99.5%, respectively, and classified to the species level of *A. baumannii*. By using the disc diffusion method, AB073 exhibited a MDRAB and a CRAB phenotype resistance to 13 drugs belonging to the 7 class of antibiotics, including penicillins (ampicillin (AMP)), *β*-lactam/*β*-lactamase inhibitor combinations (piperacillin/tazobactam (TZP) and cefoperazone-sulbactam (SCF)), cephalosporins (ceftriaxone (CRO), cefotaxime (CTX), and cefepime (CEF)), carbapenems (imipenem (IMP), meropenem (MEM), and doripenem (DOR)), aminoglycosides (amikacin (AK)), fluoroquinolones (levofloxacin (LEV) and ciprofloxacin (CIP)), and folate pathway antagonists (trimethoprim/sulfamethoxazole (SXT)). Analysis of the MIC values shows that AB073 was sensitive to two classes of antibiotics that included polymyxins (MIC of colistin: ≤1 *μ*g/ml by both broth and agar dilution methods) and tetracyclines (MIC of tigecycline: 2 *μ*g/ml). The AB073 was resistant to imipenem with an MIC of 32 *μ*g/ml.

### 3.2. General Genome Features of AB073

**Figure 1:**
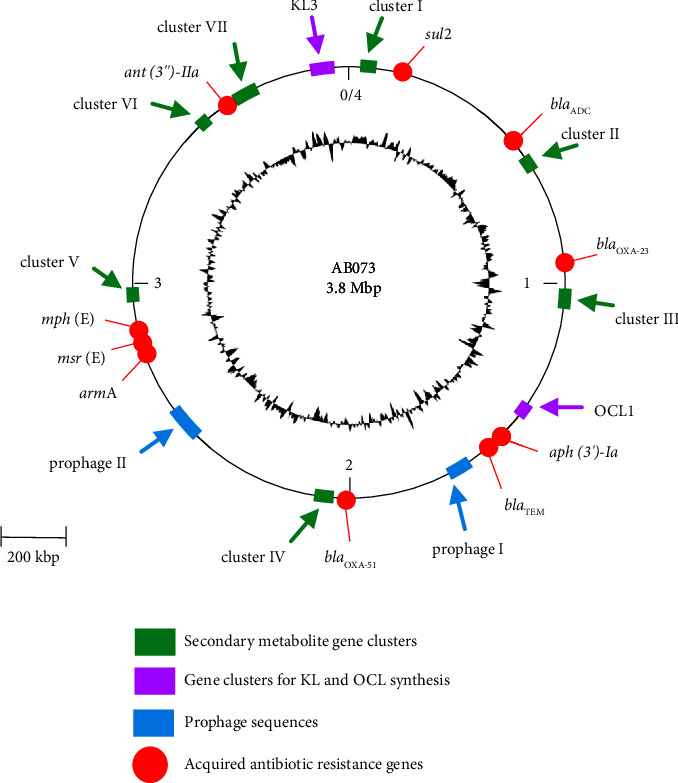
The genetic arrangement of the essential genes on the chromosome of *A. baumannii* AB073. Acquired antibiotic resistance genes, prophage genomes, locus of cell-surface polysaccharide (KL and OCL), and secondary metabolite gene clusters found in various regions on the chromosome of AB073 are labeled with various colors.

Illumina-based whole-genome sequencing produced around 12 million 151-bp paired-end reads. Using de novo assembly, we obtained 50 contigs and several contigs displayed a coverage greater than 200X (largest contig: 292,475 bp; shortest contig: 302 bp; median of contig length; 33,176 bp; and N50: 175361 bp) (Table S1). Overall, the genome size was about 3.8 Mb with a 38.95% G + C content (Table 1, Figure 1, and Table S1). Most genomes similarly related to AB073 were 6 *A. baumannii* strains which consisted of AB180-VUB, 2018BJAB2, Aba, ABCR01, 5685, and PM1912235, respectively as listed in Table 1. By using reference-guided contig ordering and orienting, all 49 assembled contigs were scaffolded into a single circular chromosome, with a total length of 3,810,754 bp (Tables 1 and S1). The remaining contig with a length of 8,731 bp could not mapped to references which was a small plasmid and identical to pPM192235_1 (CP050411) and the smallest plasmid (CP030084) was carried by the strain Aba. These plasmids were found to be the members of plasmid group 2 (GR2) belonging to plasmid lineage 1 (LN_1). Annotation of the AB073 genome revealed that the chromosome contained a total of 3,664 genes, including 3,597 protein-coding sequences (CDS), 64 tRNAs, and 3 rRNAs, while only 12 CDSs were encoded from the single small plasmid (Tables 1 and S1).

### 3.3. In Silico Detection of Resistome of AB073

**Figure 2:**
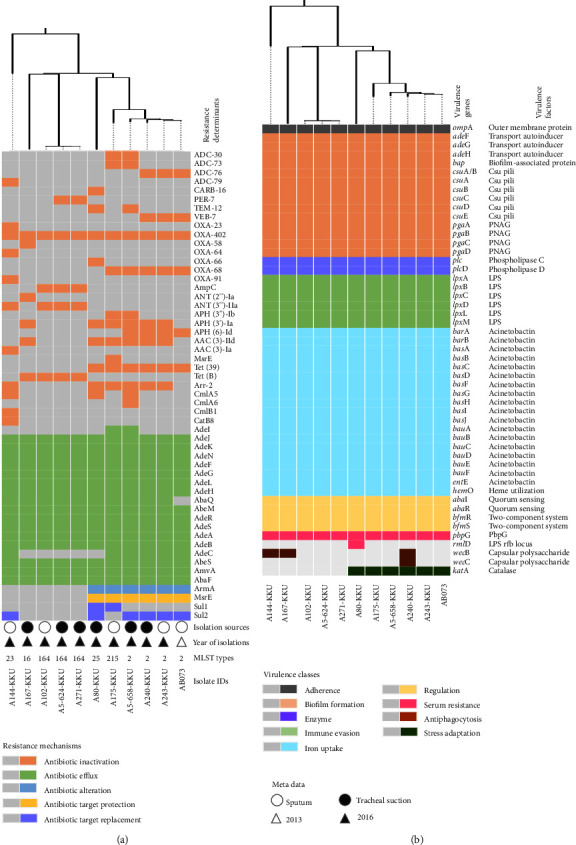
The comparative-genomic analysis of AB073 compared to additional CRAB obtained from Thailand. (a) A WGS-based phylogeny presented with a complete antimicrobial resistome and metadata of AB073 versus selected 10 CRAB isolated from Thailand. (b) A phylogenetic tree based on WGS sequence together with a complete virulome of AB073 compared to selected 10 CRAB collected from Thailand. Gray boxes represent several resistance mechanisms, virulence classes, and metadata.

Computational detection of acquired resistance genes against the ResFinder revealed that all 9 acquired antimicrobial resistance (AMR) genes, including *sul2*, *bla*_ADC_, *bla*_OXA−23_, *aph(3′)-Ia*, *bla*_TEM_, *armA*, *msr*(E), *mph*(E), and *ant(3*″*)-IIa*, were found to be located on the chromosome together with the intrinsic resistance gene known as the *bla*_OXA−51_ (Figure 1). None of the described AMR genes were detected in the 8,731 bp plasmid of AB073. We also conducted a comprehensive analysis using rMAP that combines several databases to explore the complete antimicrobial resistome and virulome of AB073 and selected 10 CRAB isolates collected from Thailand (Figure 2). Computing based on ResFinder, CARD, ARG-ANNOT, NCBI, and MEGARes annotations showed that AB073 carried additional 15 AMR determinants as illustrated in Figure 2(a). All AMR genes located on the AB073 chromosome could be classified into 5 resistance mechanisms including antibiotic inactivation, antibiotic efflux, antibiotic target alteration, antibiotic target protection, and antibiotic target replacement (Figure 2(a)). We also found putative AMR determinants involved in resistance phenotypes of penicillins (ADC-73 and TEM-12), *β*-lactam/*β*-lactamase inhibitor combinations (ADC-73 and TEM-12), cephalosporins (ADC-76, VEB-7, OXA-51/66 variant, and OXA-23), carbapenems (OXA-51/66 variant and OXA-23), aminoglycosides (APH(3′)-Ia, ArmA, AdeR, AdeS, AdeA, AdeB, and AdeC), fluoroquinolones (AdeI, AdeJ, AdeK, AdeF, AdeG, AdeL, AdeH, AbaQ, AdeM, and AbeN), and folate pathway antagonists (AdeR, AdeS, AdeA, AdeB, and AdeC). A WGS-based phylogeny revealed genetic relatedness between AB073 isolated in this work and 3 CRAB isolates belonging to a ST2-international clone 2 (Figure 2(a)). Compared to other STs (ST23, ST16, and ST164), an international clone 2 genome consisted of multiple antimicrobial resistance mechanisms such as antibiotic target alteration, antibiotic target protection, and antibiotic target replacement as presented in Figure 2(a).

### 3.4. Computational Analysis of Virulome of AB073

By using BLAST-based search, the VFDB hits contained 48 known virulence genes involved in 12 virulence factors as listed in Table 2. Among the virulence gene contents, multiple genes associated with iron uptake were identified in AB073 (*barAB*, *basABCDFGHIJ*, *bauABCDEF*, *entE,* and *hemO*). Likewise, many genes associated with biofilm formation were found such as genes that contributed to AdeFGH efflux pump (*adeFGH*), biofilm-associated protein (*Bap*), Csu pili (*csuABCDE*), and PNG (*pgaABCD*) (Table 2). There were seven known virulence genes (*lpsB* and *lpxABCDLM*) related to LPS synthesis which were responsible for immune invasion. AB073 was predicted to harbor a few genes encoded by phospholipase C and D (*plc1*, *plc2,* and *plcD*) and an outer membrane adhesin such as OmpA. The two pairs of genes including *abaIR* and *bfmRS* were identified and classified as virulence regulon genes in AB073. A single gene, *pbpG* related to serum resistance was also found to be located on the AB073 chromosome (Table 2).

On the other hand, according to the immunogenic polysaccharide typing, AB073 carried 2 loci conferring the biogenesis of lipooligosaccharide outer core (OCL1) and capsular polysaccharide (KL3) (Figure 1). We also predicted biosynthetic gene clusters and found 6 putative clusters of AB073 (cluster I to VI) related to known biosynthetic gene clusters with sizes ranging from 18.4 to 77.8 kb (Figure 1 and Table S2). Among them, cluster I was related to a redox-cofactor content, clusters II and VI appeared to be associated with siderophore productions, cluster III was found to be a beta-lactone synthesis element, and the last two clusters (IV and V) were classified as aryl polyene biogenesis operons (Table S2).

We performed a comprehensive analysis of AB073 compared to 10 CRAB isolates as illustrated in Figure 2(b). Overall, several virulence factors identified were classified as 9 virulence classes such as adherence, biofilm formation, enzyme production, immune evasion, iron uptake, regulation, serum resistance, antiphagocytosis, and stress adaptation (Figure 2(b)). Among the identified putative virulence classes, stress adaptation involved in the presence of catalase-encoded *kat*A was found in only ST2 and its relative ST25 and ST215 genomes (Figure 2(b)).

### 3.5. Genome-Based Epidemiological Analysis of AB073

**Figure 3:**
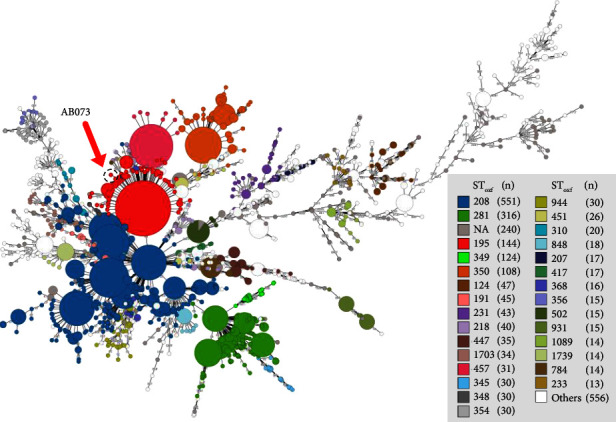
A GrapeTree clustering based on *A. baumannii* cgMLST of AB073 and other genomes deposited in the PubMLST database. Nodes differing by fewer than 7 alleles were collapsed together. Each node corresponds to a single ST_oxf_ obtained from the MLST Oxford scheme, with the diameter scaled to the number of strains and number of samples contained in them. A dashed-line circle represents the AB073 characterized in this study. A gray box illustrates the ST_oxf_ with a number of isolates.

According to the PubMLST Pasteur scheme, AB073 belonged to STpas 2 (clonal complex 2 (CCpas 2)) with the allelic profile of 2-2-2-2-2-2-2 (*cpn*60-*fus*A-*glt*A-*pyr*G-*rec*A-*rpl*B-*rpo*B). Based on the Oxford scheme, this strain was classified as SToxf 195 (CCoxf 92) and showed the profile of *glt*A-*gyr*B-*gdh*B-*rec*A-*cpn*60-*gpi*-*rpo*D as 1-3-3-2-2-96-3. Calling core genes among AB073 and other 2,647 genomes, retrieved from the PubMLST database, revealed that AB073 was found to be an international clone II (IC II) as represented in Figure 3. We also performed whole-genome SNP calculations to track the close isolates of AB073 against both complete genome and draft genome databases as shown in Tables 1 and S3, respectively. These results demonstrated that AB073-like strains could be isolated from various clinical samples such as blood, sputum, urine, and nasopharynx and are obtained from many countries such as Malaysia, China, and Belgium (Tables 1 and S3).

### 3.6. In Silico Survey of AB073 Mobilome

By using MobileElementFinder, only three insertion sequences were identified in AB073 (IS*Aba24*, IS26, and IS*Aba26*). While, searching for bacterial mobile genetic elements in oriTfinder revealed that AB073 did not carry any genes responsible for relaxase, type 4 secretion system, and type IV coupling protein. To confirm these findings, we conducted the conjugational transfer assay and failed to detect the transfer of resistance to imipenem, kanamycin, and ticarcillin between AB073 and antibiotic-sensitive recipients.

## 4. Discussion and Conclusions

Multidrug-resistant *Acinetobacter baumannii* and carbapenem-resistant *Acinetobacter baumannii* (MDRAB and CRAB) are known as successful pathogens responsible for nosocomial infections, especially for ventilator-associated pneumonia [5]. In this study, we used WGS together with phenotypic assays to retrospectively investigate the epidemiology and genomic landscape related to drug resistance and virulence factors of *A. baumannii* AB073 obtained from a patient with ventilator-associated pneumonia. Our findings corroborate the results of previous studies which reported that the most common global or international clone of carbapenemase-producing *A. baumannii* belongs to IC2 (CCoxf92/CCpas 2) in Africa, Europe, and other neighboring Asian countries such as Malaysia, China, and Vietnam [7–10, 42]. Through in-depth analysis, we found that many strains closely related to AB073 (SNP differences of less than 100) could be isolated from various clinical sources, countries, and years which tended to be successful pathogens. By comparing AB073 with a set of 10 CRAB isolates revealed that ST2-international clone 2 was the most dominant clone distributed in Thailand. On the other hand, many AB073-like strains carried two copies of *gdhB* detected by in silico analysis which was the barrier for assignation of the MLST Oxford scheme of *A. baumannii* [43].

In this work, we found that AB073 harbored many genes that contributed to multiple resistance mechanisms located on the chromosome. Major resistance mechanisms of AB073 included reduced permeability, antibiotic target protection, antibiotic target alteration, antibiotic inactivation, and antibiotic efflux. Among five putative resistance mechanisms, an antibiotic inactivation by hydrolyzing enzymes was the common pathway related to CRAB phenotype as documented previously [7]. Mobile genetic elements (MGEs), including insertion sequences (ISs), transposons (Tn), and conjugative plasmids (T4SS and T4CP) play an essential role in transferring antimicrobial resistance gene among different kinds of pathogens or between the chromosome and plasmids in a strain [44]. In contrast, we did not detect antimicrobial resistance genes embedded in MGEs of AB073. This in silico analysis was consistent with conjugation experiments, whereas AB073 does not transfer any antibiotic resistance to receipts. Moreover, in silico plasmids typing also identified only a small GR2 plasmid which is known to be an LN_1 plasmid lineage. This plasmid lineage was reported to be smaller than 10 kb in size distributed in diverse STs' *A. baumannii* strains and is nonmobilizable [33, 34]. Together, these results suggested the genetic stability of AB073 resistome.

The pathogenicity of the genome sequenced in this work and 10 genomes retrieved from the NCBI database was exacerbated by the presence of diverse genes involving a quorum sensing system, biofilm formation, iron uptake, immune invasion, toxic enzyme production, and serum resistance. Nutrient iron is known to be a key factor for *A. baumannii* growth and persistence within infection sites [45, 46]. Likewise, the strain studied in this work contained two gene clusters encoding acinetoferrins, and many genes involved in acinetobactin and heme synthesis which are important for AB073 to utilize iron. In addition, bacterial persistent invasion of the human immune system is associated with capsules, cell-surface polysaccharides, and the biofilm [47].

In conclusion, many resistance genes identified on the chromosome of AB073 could not transfer and resulted in genome stablity. However, AB073-like strains were found in different sources and countries which illustrated the ability to survive, distribute, and to be successful pathogenic strains. In support of these findings, AB073 virulome revealed the diverse key components involved in pathogenicity and successful colonizer.

## Figures and Tables

**Table 1:** General genome properties of *A. baumannii* AB073 and its relative strains.

Strain ID (country)	Sources/year of isolation (BioSample)	Chromosomal size (accession no.)	Number of plasmids	ST_pas_/ST_oxf_ (clonal complex)	No. of CDSs	No. of cgSNP^*∗∗*^
AB073 (Thailand)	Sputum/2013 (SAMN32820072)	3,815,554 bp (this study)	1 (8,731 bp)	2/195 (^*∗∗∗*^CC2)	3,585	0
AB180-VUB (Belgium)	Wound/2017 (SAMN25131651)	3,918,856 bp (CP091359.1)	ND	2/195or1816^*∗*^ (CC2)	3,653	88
2018BJAB2 (China)	Bronchial/2018 (SAMN15538706)	3,968,880 bp (CP059350.1)	1 (78,023 bp)	2/195or1816^*∗*^ (CC2)	3,728	229
Aba (China)	Sputum/2016 (SAMN09389171)	3,911,735 bp (CP030083.1)	1 (8,732 bp)	2/195or1816^*∗*^ (CC2)	3,640	290
ABCR01 (Thailand)	Sputum/2019 (SAMN12569102)	4,030,371 bp (CP042931.1)	ND	2/195or1816^*∗*^ (CC2)	3,847	323
5685 (China)	Blood/2016 (SAMN24114662)	3,952,283 bp (CP096734.1)	ND	2/195or1816^*∗*^ (CC2)	3,701	975
PM1912235 (India)	Pus//2019 (SAMN14420246)	3,926,441 bp (CP050410.1)	1 (8,731 bp)	2/195or1816^*∗*^ (CC2)	3,654	1070

ND, not detected; STpas, MLST Pasteur scheme; SToxf, MLST Oxford scheme; CC, clonal complex. ^*∗*^Two copies of *gdhB* were detected by in silico determination based on the MLST Oxford scheme. ^*∗∗*^Number of core-genome SNPs compared to AB073. ^*∗∗∗*^Clonal complex 2.

**Table 2:** Computational identification of genes encoding for virulence factors of AB073.

Virulence factors	Potential virulence classes	Related virulence genes and operons
Acinetobactin	Iron uptake	*barAB*, *basABCDFGHIJ*, *bauABCDEF,* and *entE*
Heme utilization	Iron uptake	*hemO*
AdeFGH efflux pump	Biofilm formation	*adeFGH*
Biofilm-associated protein	Biofilm formation	*Bap*
Csu pili	Biofilm formation	*csuABCDE*
PNAG	Biofilm formation	*pgaABCD*
LPS	Immune invasion	*lpsB* and *lpxABCDLM*
Phospholipase C and D	Enzyme production	*plc1*, *plc2*, and *plcD*
Outer membrane protein	Adherence	*ompA*
Quorum sensing	Regulation	*abaIR*
Two-component system	Regulation	*bfmRS*
pbpG	Serum resistance	*pbpG*

PNAG, polysaccharide poly-N-acetylglucosamine.

## Data Availability

The data used to support the findings of the study are included within the article.

## Supplementary Materials

Supplementary Materials Table S1: standard genome characteristics of the *Acinetobacter baumannii* AB073 resulted from de novo genome assembly and reference-based ordering; Table S2: secondary metabolite gene clusters found in the *A. baumannii* AB073 genome predicted by antiSMASH v6.0.; and Table S3: in silico identifications of AB073-like strains retrieved from the databases.
