# Supplementary material for: Genomic Landscape Reveals Chromosomally-Mediated Antimicrobial Resistome and Virulome of a High-Risk International Clone II Acinetobacter baumannii AB073 from Thailand
Source: Glob Health Epidemiol Genom. 2024 Apr 30;2024:8872463. doi: 10.1155/2024/8872463 (PMC11074871; doi:10.1155/2024/8872463)
Supplement: Supplementary Materials — Table S1: standard genome characteristics of the Acinetobacter baumannii AB073 resulted from de novo genome assembly and reference-based ordering; Table S2: secondary metabolite gene clusters found in the A. baumannii AB073 genome predicted by antiSMASH v6.0.; and Table S3: in silico identifications of AB073-like strains retrieved from the databases. [file 8872463.f1.docx]

**Table S1:** Standard genome characteristics of the *Acinetobacter baumannii* AB073 resulted from de novo genome assembly and reference-based ordering

| **Feature** | **Genome** | **Chromosome** | **Plasmid** |
| --- | --- | --- | --- |
| Total number of bases | 3,819,485 bp | 3,810,754 bp | 8,731 bp |
| Guanine-Cytosine (GC) content | 38.95 % | 38.91 % | 34.37 % |
| No. of total contigs | 50 contigs | 49 contigs | 1 contig |
| L50 | 9 contigs | NA | NA |
| N50 | 175,361 bp | NA | NA |
| L90 | 57,136 bp | NA | NA |
| N90 | 21 contig | NA | NA |
| Length of longest contig | 292,475 bp | NA | NA |
| Length of shortest contig | 302 bp | NA | NA |
| Median Length of all contigs | 33,176 bp | NA | NA |
| Number of scaffolds (after reference-based ordering) | ND | 1 | 1 |
| No. of tRNAs | 64 tRNAs | 64 tRNAs | ND |
| No. of rRNAs | 3 rRNAs | 3 rRNAs | ND |
| No. of Coding sequence (CDS) | 3,597 CDS | 3,585 CDS | 12 CDS |
| Replicon type | DnaA and GR2 or LN_1 ^a^ | DnaA | GR2 or LN_1 ^a^ |

NA; Not analyzed, ND; Not detected

^a^ Classification of plasmid replication types, GRs and LNs, were conducted as previously reports (Bertini et al., 2010; Salgado-Camargo et al., 2020).

**Table S2:** Secondary metabolite gene clusters found in the *A. baumannii* AB073 genome predicted by antiSMASH v6.0.

| **Cluster no.** | **Types** | **Cluster length** | **Genome position** | **Most similar known cluster** | **MIBiG accession no.** | **Putative encoded compounds** |
| --- | --- | --- | --- | --- | --- | --- |
| I | redox-cofactor | 22.1 kb | 658,789 to 680,979 | lankacidin C biosynthetic gene cluster of *Streptomyces rochei* (13% similarity) | BGC0001100 | lankacidin C |
| II | siderophore | 40.2 kb | 1,070,178 to 1,110,461 | Acinetoferrin biosynthetic gene cluster of *A. haemolyticus* (30% similarity) | BGC0000295 | acinetoferrin |
| III | betalactone | 29.0 kb | 2,172,536 to 2,201,617 | mycosubtilin biosynthetic gene cluster of *Bacillus subtili*s ATCC 6633 (20% similarity) | BGC0001103 | mycosubtilin |
| IV | arylpolyene | 33.9 kb | 2,913,469 to 2,947,467 | berninamycin A biosynthetic gene cluster of *Streptomyces bernensis* (26% similarity) | BGC0001472 | berninamycin A |
| V | arylpolyene | 18.4 kb | 3,508,311 to 3,526,754 | APE Vf biosynthetic gene cluster of *Aliivibrio fischeri* ES114 (45% similarity) | BGC0000837 | APE Vf |
| VI | siderophore | 77.8 kb | 3,652,445 to 3,730,306 | Acinetoferrin biosynthetic gene cluster of *A. haemolyticus* (20% similarity) | BGC0000295 | acinetoferrin |

**Table S3:** In silico identifications of AB073-like strains retrieved from the databases.

| **IDs** | **Accession number** | **SToxf** | **Isolation Source** | **Countries isolated** | **Year of isolations** | **No. of SNPs distance** |
| --- | --- | --- | --- | --- | --- | --- |
| AC38 | VZZP01 | 195 | ETT secretion | Malaysia | 2011 | 89 |
| AB22 | WBIZ01 | 195 | Pus | Malaysia | 2012 | 79 |
| AB2 | WBJJ01 | 195 | urine | Malaysia | 2012 | 83 |
| LY3 | JDSX01 | 195 | NR | China | 2012 | 83 |
| AB5 | WBJH01 | 195 | Pus | Malaysia | 2012 | 87 |
| AB18 | WBJC01 | 195 | blood | Malaysia | 2012 | 90 |
| AB13 | WBJE01 | 195 | ETT secretion | Malaysia | 2012 | 91 |
| AB8 | WBJF01 | 195 | blood | Malaysia | 2012 | 91 |
| AB20 | WBJB01 | 195 | Pus | Malaysia | 2012 | 93 |
| GZ2012037 | VKKC01 | 195 | bloodstream | China | 2012 | 96 |
| 461 | LCTE01 | 195 | Wound swab | Malaysia | 2013 | 84 |
| AB_HZ_S93 | PRGO01 | 195 | sputum | China | 2013 | 95 |
| 4300STDY7045748 | UFJS01 | 195 | NR | Thailand | 2016 | 91 |
| MDR_CQ | CP019114 | 195 | NR | China | 2016 | 93 |
| 4300STDY7045899 | UFPY01 | 195 | NR | Thailand | 2016 | 94 |
| 4300STDY7045747 | UFJV01 | 195 | NR | Thailand | 2016 | 95 |
| MDR_CQG | SNVN01 | 195 | NR | China | 2017 | 83 |
| MDR_CQJ | SNVK01 | 195 | NR | China | 2017 | 86 |
| MDR_CQC | SNVR01 | 195 | NR | China | 2017 | 87 |
| SCPM_O_B_8565_4533_ | VBXM01 | 195 | sputum | NR | 2017 | 92 |
| WCHAB090203 | SGTS01 | 195 | NR | China | 2017 | 93 |
| SCPM_O_B_8567_4586_ | VBXO01 | 195 | sputum | NR | 2017 | 94 |
| CAb26 | VCOE01 | 195 | Bronchoalveolar | China | 2018 | 96 |
| NSAb26 | VCOF01 | 195 | nasopharynx | China | 2018 | 98 |

NR; Not reported

* The difference of SNPs smaller than 100 was considered as positive cutoff.
